# Thromboelastography platelet mapping for surgical patients: lessons from cardiac surgery

**DOI:** 10.1007/s10195-013-0284-5

**Published:** 2013-12-29

**Authors:** Priyadharshanan Ariyaratnam, Lindsay A. Mclean, Kostas Kotidis, Mahmoud Loubani

**Affiliations:** Department of Cardiothoracic Surgery, Castle Hill Hospital, Cottingham, HU16 5JQ UK

Dear Editor,

It was with great interest that we read the letter by Callender et al. [1], describing the use of thromboelastography (TEG) and thromboelastography platelet mapping (TEG-PM) in patients undergoing hip replacements whilst still on antiplatelet medication.

TEG is a highly efficient system for discerning clotting abnormalities in a setting where time is of the upmost importance [2]. The many disciplines involved in the management of the acute cardiac surgical patient, including perfusionists and anaesthetists, have a high appreciation of the device [3].

There is little point in having a screening test, as in the case of TEG-PM, without a treatment strategy in place. If a patient was found to have significant inhibition of their platelets, what does one do? One strategy would be to wait until the clotting function normalised, and this would be appropriate in the case of elective cases where antiplatelet treatment can be stopped a few days prior. Callender’s letter was itself a response to Hossain et al. [4] describing patients on antiplatelet therapy who were undergoing repair of hip fractures. Hip fractures require urgent surgery but one can argue that in patients that are stable, the operation can be delayed until platelet function normalises after discontinuation of anti-platelet medication. Hossain et al. have also shown that these patients may not be at an increased risk of bleeding anyway.

However, what happens in the case of emergencies as in the scenario of significant trauma? Our experience is somewhat peculiar in this regard.

We in cardiac surgery have developed a protocol of giving platelets prophylactically during the operation in patients with more than 70 % platelet inhibition and where surgery cannot wait.

It is a bold move to make given that platelet transfusion is not without its risk and is certainly not cheap.

However, recent evidence shows that there is little negative impact in giving platelets peri-operatively in patients with a high platelet inhibition on TEG-PM. In fact, it improves the clotting profile very quickly [5]. Despite this, the results for the impact on post-operative bleeding and product transfusion are still pending.

Hence, TEG-PM has the potential to be a very nifty rapid analysis tool not only for predicting surgical patients at an increased risk of bleeding, as in the case of patients on antiplatelet therapy, but also to implicate a treatment strategy in urgent patients. To err on the side of caution, however, there must be a greater impetus on randomised clinical trials of such a treatment strategy across all modalities of surgery, including orthopaedics and trauma, since the physiology and clotting changes that occur during cardiac surgery do not necessarily occur during general orthopaedic surgery.
